# Media Exposure of Suicidal Behaviour: An Umbrella Review

**DOI:** 10.3390/nursrep13040125

**Published:** 2023-10-25

**Authors:** Teresa Sufrate-Sorzano, Marco Di Nitto, María Elena Garrote-Cámara, Fidel Molina-Luque, José Ignacio Recio-Rodríguez, Pilar Asión-Polo, Ángela Durante, Vicente Gea-Caballero, Raúl Juárez-Vela, Jesús Pérez, Iván Santolalla-Arnedo

**Affiliations:** 1 Care and Health Research Group, GRUPAC, Nursing Deparment, University of La Rioja, 26006 Logroño, Spain; teresa.sufrate@unirioja.es (T.S.-S.); maria-elena.garrote@unirioja.es (M.E.G.-C.); ivan.santolalla@unirioja.es (I.S.-A.); 2 Biomedical Research Centre of La Rioja, CIBIR, 26006 Logroño, Spain; 3 Department of Health Sciences, University of Genoa, 16126 Genova, Italy; marco.dinitto@unige.it; 4 Faculty of Education, Psychology and Social Work, University of Lleida, 25001 Lleida, Spain; fidel.molinaluque@udl.cat; 5 Group for the Study of Society, Health, Education and Culture (GESEC), University of Lleida, 25001 Lleida, Spain; 6 Research Institute in Social and Territorial Development (INDEST), University of Lleida, 25001 Lleida, Spain; 7 Faculty of Nursing and Physiotherapy, University of Salamanca, 37008 Salamanca, Spain; donrecio@usal.es; 8 Primary Care Research Unit of Salamanca (APISAL), Institute of Biomedical Research of Salamanca (IBSAL), 37008 Salamanca, Spain; 9 Aragonese Health Service (SALUD), 50017 Zaragoza, Spain; piasion@unirioja.es; 10 Department of Translational Medicine, University of East Piedmonet, 13100 Vercelli, Italy; angela.durante@uniupo.it; 11 Faculty of Health Sciences, International University of Valencia, 46002 Valencia, Spain; vagea@universidadviu.com; 12 Prevention and Early Intervention in Mental Health (PRINT), Institute of Biomedical Research of Salamanca (IBSAL), 37008 Salamanca, Spain; jesusperez@usal.es; 13 Faculty of Medicine, University of Salamanca, 37008 Salamanca, Spain; 14 Department of Psychiatry, University of Cambridge, Cambridge CB2 1TN, UK

**Keywords:** communications media, general literature review, Papageno effect, prevention, suicide, suicidal behaviours, suicidal ideation and Werther effect

## Abstract

Aim: To analyse recommended interventions for the safe and responsible dissemination of suicidal behaviour in the media for preventive purposes. Background: Suicide is a serious public health problem that leads to more than 700,000 deaths per year, which translates into one death every forty seconds. The media play a significant role in shaping public perceptions and reflecting societal issues. Because of its active role in the construction of reality, the way in which the media report and expose suicidal behaviour has the capacity to influence the population in either a preventive or harmful way. Design: An umbrella review was carried out and a report was written according to the Preferred Reporting Items for Overviews of Reviews. Methods: We systematically searched for reviews published from inception to February 2023 in MEDLINE (PubMed), CINAHL and PsycInfo (via EBSCOhost), Web of Science, Embase, Cochrane Library of Systematic Reviews, Scopus, and Google Scholar. A narrative synthesis of the results was conducted. Results: Six systematic reviews with a moderate to high quality level were selected. Among the recommended interventions were the inclusion of positive messages of hope, resilience, or of overcoming the event, narratives with information on available resources or the promotion of support-seeking attitudes as an effective prevention mechanism, as well as the avoidance of repetitive reporting of the same suicide. The appropriate and responsible dissemination of information on suicidal behaviour in the media with complete and up-to-date information on available centres, organisations, institutions, and resources has proven to be effective, especially in vulnerable populations. Conclusion: Educating and training the media in an appropriate approach to disseminating suicidal behaviour helps to reduce the number of suicidal behaviours. Knowing what information is advisable to include in the news item as well as what information to avoid is a strong point. Guidelines to promote responsible media reporting are a key component of suicide prevention strategies. This study was prospectively registered in the International Prospective Register of Systematic Reviews (PROSPERO) on 23 April 2022 with the registration number CRD42022320393.

## 1. Introduction

In 1976, the World Health Organization (WHO) defined the term suicide as “*an act with a lethal outcome, deliberately initiated and carried out by the subject, knowing or expecting its lethal outcome and through which they intend to obtain the desired changes*” [1]. It is known that the first suicidal behaviour dates to prehistoric times. The social conception of this universal phenomenon has changed according to the cultural, religious, and intellectual principles of history. Thus, the same lethal act has been accepted in some cultures as a transition to a new immortal stage, and in others, it has been punished and penalised as a crime [2]. Human beings have redefined what suicide represents in each historical context, and although the approaches have been disparate, no one has been indifferent to this phenomenon [3].

Suicide is now recognised as a serious public health problem due to the fact that there are more than 700,000 deaths per year, which translates into one death every forty seconds, and for every suicide, an estimated twenty attempts are made [4]. In addition, the psychological impact of suicidal behaviour (suicide ideation, attempt, and death by suicide) on close, personal circles, affecting, on average, six suicide loss survivors, is also highly significant [5]. Furthermore, it is currently estimated that an average of 100 community members may be affected after each suicide [6,7]. For these reasons, both the United Nations Sustainable Development Goals and the WHO’s Comprehensive Mental Health Action Plan 2013–2030 have set a target of reducing these figures by one-third [8,9].

Suicidal behaviour is a complex phenomenon caused by a multitude of biological, psychological, social, cultural, and environmental factors which are associated with situations of crisis, stress, or traumatic moments that have not been dealt with, triggering suicide as an escape route [10]. Prevention is possible, and this requires the collaboration and coordination of different multidisciplinary teams that are committed to providing integrated and holistic care, as an individual approach is not effective in such a complex process [11].

Among the various health professionals needed to tackle the problem, the generalist nurse, and, specifically, the mental health specialist, play key roles in prevention. Their functions include treating underlying mental disorders and the identification and assessment of populations in situations of vulnerability, controlling environmental risk factors and stressful life events, identifying alcohol withdrawal, limiting access to the resources most frequently used for suicide, breaking the socio-cultural stigma that prevents access to mental health services, and promoting community health education as a highly vital method for the responsible, safe, and useful dissemination of information through the media [12].

The media play a significant role in shaping public perceptions and reflecting societal issues by allowing the transmission of information between sender and receiver; specifically, when the receiver is a social group, they are called Mass Media. Among the most prominent media are radio, internet, and television, whose news influences people’s thoughts, values, and actions on political, economic, and social issues. Therefore, the media play an active role in society due to their direct influence on the way reality is perceived [13].

This approach is based on the theory of agenda-setting, which explains how the media are the most notable factor in the social construction of everyday reality so that the issues dealt with in the media will become the issues of greatest concern to society by directing attention and changing the way people think about them [14]. Therefore, the way in which the media report and expose suicidal behaviour can have a preventive or protective influence on suicidal behaviour or, conversely, a detrimental influence by causing an increase in numbers through contagion or imitation.

The origin of the Werther effect alludes to Goethe’s novel “The Sorrows of Young Werther”, where the protagonist takes his own life by shooting himself. After its publication, a wave of young people died by suicide using the same method, wearing the same clothes as the character, and making references to the work in their suicide notes [15,16,17]. Some authors propose that this effect may have originated earlier with William Shakespeare’s Romeo and Juliet, as it caused a multitude of deaths among those unlucky in love. However, it was the sociologist Phillips in 1974 who framed this effect, stating that the more suicide was portrayed in the media, the higher the suicide figures were later on [15,16].

The basis of the protective effect, also called Papageno, aims to repeal the existing taboo on suicide by relying on responsible and truthful communication [16]. Such communication should meet established criteria such as the use of clear and understandable terminology, informing about the preventable nature of suicide, providing helpline numbers, informing about the link between suicide and depression, emphasising that it is a treatable condition, respecting the privacy of affected families, raising awareness among the general public so that they can be aware of risk indicators in the immediate environment, and providing information about support services and prevention programmes [17,18,19].

The Werther effect has repeated itself on several occasions throughout history; a recent media example is the broadcast of the series “13 Reasons Why” in March 2017 which explicitly and graphically showed the suicide of the teenage protagonist. Specifically, between March and April 2017, there were 1.5 million searches related to suicide on Google, with the most frequently searched phrases being “*how to slit your wrists*”, “*how to commit suicide*”, and “*how to kill yourself*” [20]. In 2019, research by Niederkrotenthaler et al. found an increase in suicides in the three months following the premiere of the series, higher than the general trend, among 10–19-year-olds and especially in females [21].

Frequently, media reports exaggerate the most tragic, lethal, and unusual methods, such as using a firearm or jumping onto railway tracks. These methods do not often correspond to the reality in most countries, where hanging is more common [22]. In this umbrella review, the concept of intervention is used to refer to the mode of media exposure and dissemination of suicidal behaviour. For that, we aimed to analyse the recommended interventions for the safe and responsible reporting of suicidal behaviour in the media for preventive purposes.

## 2. Materials and Method

This is an umbrella review conducted according to the Joanna Briggs Institute (JBI) methodological manual [23]. This systematic approach is guided by providing a comprehensive and objective synthesis through the use of rigorous and transparent methods. A preliminary search was conducted on PubMed to identify existing systematic reviews that met the inclusion criteria. From this preliminary search, several systematic reviews potentially falling within the inclusion criteria were found, which justified the use of an umbrella review for the purpose of the study. The report was written according to the Preferred Reporting Items for Overviews of Reviews (PRIOR) statement [24]. The protocol of this umbrella review was registered in the International Prospective Register of Systematic Reviews (PROSPERO) with registration code CRD42022320393.

Ethical considerations related to the review process: None of the data presented in this paper have been plagiarised, invented, manipulated, or distorted.

### 2.1. Search Strategy

A systematic search was conducted for systematic reviews published from inception to the 13 February 2023. Eight databases were consulted: MEDLINE (PubMed), CINAHL and PsycInfo (via EBSCOhost), Web of Science, Embase, Cochrane Library of Systematic Reviews, Scopus, and Google Scholar.

The search terms that guided the search were: suicide, “suicide, completed”, “suicidal ideation”, “suicide, attempted”, “audiovisual aids”, radio, television, telecommunications, and “systematic review”. The search was first performed in PubMed, applying Mesh terms, free text terms, and using wildcards if deemed appropriate. Then, the final search was tailored for use in all the other databases considered. The complete search strategy can be found in Supplementary File S1.

### 2.2. Inclusion and Exclusion Criteria

We included all systematic reviews written in English, Spanish, or Italian regarding the dissemination of news in the communication media with the aim of reducing suicidal intentions. Primary studies, books, book sections, and grey literature (theses, conference proceedings, etc.) were excluded, as well as systematic reviews that did not focus on our topic.

### 2.3. Outcome Measures

The primary outcome of this umbrella review was suicide rate reduction following news dissemination. The secondary outcomes considered were the number of suicides and suicidal behaviour reduction.

### 2.4. Review Selection

All studies were retrieved from each database and were uploaded to a Microsoft Excel^®^ (Version 16.66.1) spreadsheet and duplicates were removed. The Excel^®^ tool was used to manage data collection, facilitating the organisation of information by title, journal, database, keywords, abstract, or year. Titles and abstracts were screened by two separate authors (Author 1 and Author 2) in accordance with the criteria for eligibility. After the preliminary phase, they separately evaluated the full texts of studies that might be pertinent for inclusion.

Any discrepancies were resolved by discussion between the authors, and when consensus was not reached, a third researcher (Author 3) was consulted.

### 2.5. Quality Assessment

The methodological quality of the selected papers was analysed independently by two reviewers (Author 1 and Author 2) (Table 1). When a consensus was not reached, a third researcher (Author 3) participated in the quality assessment. For this purpose, the Critical Appraisal Skills Programme Spanish (CASPe) tool was used in its version for systematic reviews [25]. The checklist consists of ten items designed to assess quality and considers three broad areas when evaluating a systematic review: are the results valid; what are the results; will the results help locally? The research team considered that if there was at least one response scored as “no” or “unclear” on one of the ten items, a moderate quality of the review would be inferred. If there were at least three “no” or “unclear” responses, it would be defined as a low-quality review. In the presentation of the results and the generalisation of the results, the reported quality was considered. The results obtained are shown in Table 1.

### 2.6. Data Extraction

Following the screening phase, two authors (Author 1 and Author 2) separately collected and extracted all data using a standard data collection form regarding systematic review characteristics: reference and year, general objective, review typology, databases, the period covered, and outcome data. Conflicts were resolved by consultation with a third reviewer (Author 3).

### 2.7. Data Synthesis

According to the JBI methodological manual [23], which emphasises that the results of an umbrella review are reported to provide existing research syntheses relevant to a particular topic, the data of the included systematic reviews were summarised in narrative form. The results were presented both in the form of a table and within the text.

## 3. Results

**Figure 1:**
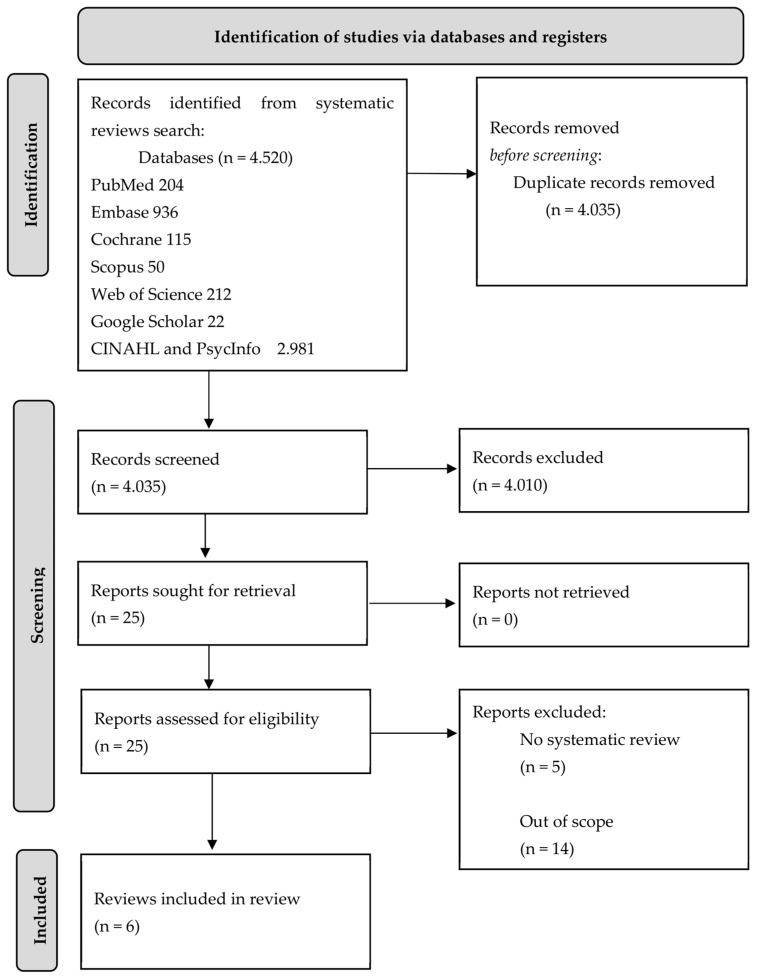
PRIOR flow diagram.

The search collected a total of 4520 articles (PubMed 204, CINAHL and PsycInfo 2981, Web of Science 212, Embase 936, Cochrane 115, Scopus 50, and Google Scholar 22). After the removal of duplicates, 4035 results were reviewed by title and abstract to assess relevance and eligibility criteria, eliminating 4010 records and including 25 papers. Finally, after eliminating 19 papers (n = 5 were not considered truly systematic reviews due to their methodology and n = 14 did not answer the research question posed for this umbrella review), 6 systematic reviews were analysed for the development of this research. Of the included reviews, three also included meta-analyses. The time range covered by the final reviews is from 2005 to 2022. The procedure followed in this umbrella review is described in Figure 1.

### 3.1. Methodological Quality Assessment

In relation to the assessment of methodological quality, three reviews were inferred to be of high quality [26,27,28] and three of moderate quality [29,30,31]. All reviews were included in the umbrella review as their results could be generalised and applied to the population. The main findings are presented as a narrative synthesis.

### 3.2. Characteristics of the Included Studies

The literature search dates of the included reviews ranged from the inception of the database to 2021. The reviews included a total of 195 unique primary studies. Descriptive observational designs were the most frequent primary study type (n = 136), followed by randomised controlled trials (n = 29), analytical observational studies (n = 25), and quasi-experimental studies (n = 5). A median of five primary studies (inter-quartile range 3–7) were included.

In the reviews included, the strategies or tools recommended being implemented by the media for the responsible and safe dissemination of suicidal behaviours to the population were considered as an intervention, either by adding certain aspects (n = 9) or by eliminating certain characteristics related to the suicidal behaviour described (n = 4).

A summary of the general characteristics of the included reviews is reported in Table 2.

### 3.3. Overlap between Included Systematic Reviews and Studies

The primary studies included across the systematic reviews and relevant to the aim of this study were mapped and the overlap among all reviews was analysed. Only one primary study overlapped with another review. A total of thirty primary studies were cited thirty-one times across the six systematic reviews included in this overview, resulting in an overall corrected covered area (CCA) of 0.07, indicating almost no overlap across the included reviews. In Table 3, it can be seen that only one of these primary studies overlaps, i.e., it was analysed in two of the systematic reviews. This result is considered positive for the research as it infers that the included reviews did not analyse the same primary studies [32] (Table 3).

### 3.4. Summary of Evidence

For a better analysis and to facilitate understanding, the results are presented in terms of the aspects or characteristics that the media should include in the information dissemination related to suicidal behaviour and those that should be avoided, all with the ultimate aim of developing responsible dissemination based on prevention.

### 3.5. Recommended Strategies to Be Included in Responsible Dissemination

With regard to the strategies that are recommended to be included in the dissemination and found to be most widely represented in the reviews, the first is the inclusion in the narrative of positive messages of hope, resilience, and overcoming adversity [26,27,28,30,31], finding a protective effect of up to one month’s duration in vulnerable populations [26]. Subsequently, the promotion and encouragement of the search attitude as an effective care tool for the general population are present in three studies [27,29,30]. In this line, the effectiveness of such an intervention when specifically focused on the male gender is worth highlighting [26,29]. With regard to the detrimental effect or Werther effect of developing suicidal behaviour following media reports, the work of Sisask et Värnik shows a strong association with age and gender, making young and old people more vulnerable to the imitation effect [30].

The inclusion in the narrative of complete and up-to-date information on available facilities, organisations, institutions, and resources has been shown to be effective [26,29,31]. This exposure includes providing the population with complete information, including contact telephone numbers or updated website addresses.

The media awareness of mental health and mental disorders [29,31] along with the dissemination of treatment availability, especially for depression, have also proven to be effective in the responsible and preventive dissemination of suicidal behaviour in the media [29]. Along these lines, there is a reference to awareness-raising as a method for literacy and public awareness in order to decrease the stigma of mental health in general and suicide in particular [29].

In the reviews analysed, some strategies focus on the contribution that health professionals and survivors of suicide can make to society, and community involvement has been shown to be critical to success [29]. Survivors are understood as those people negatively and significantly affected by the suicide of someone around them or those people who have faced a suicide attempt, highlighting, as a strategy in suicidal ideation, the dissemination of real personal stories and the sharing of stories that reflect overcoming or recovering from suicidal crises [26]. Regarding the specific role of healthcare professionals, the work of journalists or scriptwriters with experts in the field of mental health is effective for safe exposure [27]. Narratives that include the idea that suicide prevention is possible are also effective [29].

### 3.6. Strategies to Avoid Responsible Dissemination

In this section, as mentioned above, the strategies found in the included reviews that should be avoided for the dissemination and safe exposure of suicidal behaviour will be analysed. The omission of an explicit description of the method used and the location where the suicidal behaviour took place stands out in most of the works [27,28,30,31]. This strategy is based on the correspondence found between the subsequent increase in suicide rates following the media coverage of suicidal behaviour related to the explicit dissemination of the method used [29]. Along these lines, narratives that avoid a romantic, dramatic, or glorified depiction of suicidal behaviour have also been shown to be effective [28,30,31].

Not repeatedly reporting the same behaviour and not depicting suicide as an inevitable event with no option to intervene in prevention have also been shown to be effective interventions [28]. One response to these facts may lie in the bystander normalisation of suicidal behaviour as a quick escape route or solution to problems. In Table 4, the interventions found in the review are represented chronologically.

## 4. Discussion

This is the first general review that explores the media as a key interpersonal and social factor. The media may function as a protective and/or risk factor for suicidal behaviour because it plays an active role in society by directly influencing the way reality is perceived. The way in which the media report and expose information related to suicidal behaviour is decisive. Well-managed information or exposure has a preventive influence in reducing suicide rates, while poor media reporting can lead to an increase in numbers through contagion or imitation. A broad search strategy has been used to ensure a comprehensive synthesis of the systematic reviews in this area, providing an integrated and comprehensive overview of a high level of evidence. The assessment of the methodological quality of the included systematic reviews, conducted by three independent reviewers, determined a high-quality rating for the majority of studies.

The results confirm that the inclusion in the narrative of positive messages of hope, resilience, and overcoming adversity is present as preventive and protective information in the most current systematic reviews published between 2020 and 2022 [26,27,28]. Establishing the adequate and responsible dissemination of suicidal behaviour in the media with complete and updated information on the centres, organisations, institutions, and resources available for dealing with suicidal behaviour has proven to be effective, especially in vulnerable populations and/or those with difficulties in accessing the health system [12,26,29,31]. The use of the media as a health literacy tool, reducing the stigma of mental illness in general and suicidal behaviour in particular, as well as the dissemination of available treatments, have been shown to be a preventive strategy in several studies [29,31]. Silencing suicide does not contribute to reducing the number of suicidal behaviours, but rather causes continuous stigmatisation of the event and its consequent consideration as a taboo subject by society [33]. The figure of the gatekeeper, understood as the person who acts as an information specialist, in the media is key to the prevention of suicidal behaviour, working in not only a *reactive* capacity, i.e., responding effectively to the demands for information that they receive, but also *proactively*, i.e., anticipating information needs before they are perceived [14]. Promoting the figure of the gatekeeper is a line of action to develop competencies for the prevention of suicidal behaviour in social agents who are in direct contact with the population [11]. The work of gatekeepers in the media for the protection of mental health and reduction in suicidal ideation and attempts can be carried out by health professionals, mental health nurses, psychologists, psychiatrists, etc., as well as by survivors or patients who tell personal stories of overcoming suicide; community participation has been shown to be fundamental for preventive success, with the key being to show suicide prevention as possible in the media [27,29]. The results of this umbrella review are in line with existing recommendations in the field and along the lines of providing well-managed information for suicide prevention. The WHO recommends providing accurate information on where to seek help; educating the community about the facts of suicide and suicide prevention, without spreading myths; and disseminating stories about how to cope with life stressors or suicidal thoughts, and how to obtain help [17]. The Action Alliance in its 2022 report suggests preventively working with mental health experts to ensure safe outreach and exposure; using non-judgmental language; and providing narratives with information on available facilities, organisations, and resources (including up-to-date contact numbers or websites) [34].

Following a review of the scientific literature, it can be determined that certain media interventions may become risk factors for suicidal behaviour. With regard to the placement of news about suicide, the Canadian Psychiatric Association and the Canadian Association for Suicide Prevention state that news about suicidal behaviour should not be displayed on the front or back page of newspapers, should avoid sensationalism, should not provide details about the site/location, should not explicitly describe the medium used, should not use photographs, video footage, or social media links, and should not repeat the news story unduly [35,36]. This is in line with the WHO, which has worked on several manuals to approach this issue, and their recommendations [17,37,38]. In 2017, an observational study by Acosta-Artiles et al. showed that the press publishes news in an unjustified manner, one-third of which is avoidable; it does not provide new information and it contains a high percentage of characteristics that are harmful to viewers, which may increase the risk of contagion. It should be taken into consideration that not only the quality of dissemination has an influence, but also the quantity [39]. In research by Armstrong et al., semi-structured interviews were conducted with media professionals in India who had previously published news about suicides, and several participants stated that violent and novel methods of suicide were of great interest to the press [40]; media education and intervention at this level is essential to prevent the dissemination of this type of sensationalist information which is a high-impact risk factor. The Action Alliance’s 2022 report along these lines recommends not explicitly describing the method used and not reducing the multi-causality of suicidal behaviour to a single precipitating factor or a simple explanation [34].

In Spain, there is no suicide prevention plan at the national level on which to contrast results. In some autonomous communities, the Suicide Prevention Plan is included within a Strategic Mental Health Plan, a document that addresses the general objectives and interventions to be developed within mental health, but not expressly for suicide [41]. Specifically, in the community of La Rioja, there is a Suicide Prevention Plan. This document includes a specific section related to the prevention of suicidal behaviour and the media [42].

The results of this review are in line with this regional plan where the training of communication professionals and the development of style guides are indicated as effective interventions for the dissemination of suicidal behaviour.

## 5. Limitations

A possible limitation of the study is that if alternative search commands were used, additional studies might have been found. However, the authors believe that if the search procedure is modified, the conclusions may be largely the same, so this may not be such a serious limitation.

The focus of this umbrella review on suicidal behaviour and the media was on news descriptions and their dissemination. However, research on the specific representation of suicide in films, series, performing arts, or other forms of social dissemination could be of great interest.

Similarly, the number of studies included and the moderate quality in three of them can also be considered a limitation.

## 6. Implication for Practice

Although psychiatric disorders significantly increase the risk of suicide, interpersonal and social factors also play an important role. The media is a feature of the social environment in which suicidal behaviour can be learned; though the effect is probably smaller than that of other psychosocial risk factors for suicide, it is a significant agent in the social construction of reality, especially for vulnerable people [43].

## 7. Conclusions

Evidence confirms that suicide is preventable and that the comprehensive coordination of different multidisciplinary teams is necessary to be effective in suicide prevention. Educating and training the media in the appropriate approach for disseminating suicidal behaviour helps to reduce the number of suicidal behaviours. Knowing what information is advisable to include in the news item as well as what information to avoid is a strong starting point. Guidelines to encourage the responsible reporting of suicide in the media are a key component of suicide prevention strategies. Multidisciplinary health teams in collaboration with the media could be helpful in ensuring prevention-based outreach.

## Figures and Tables

**Table 1:** Methodological quality analysis tool for systematic reviews using CASPe. Each criterion is scored as yes, no, or unclear.

			Authors			
Items	Niederkrotenthaler et al., 2022 [26]	Niederkrotenthaler et al., 2021 [27]	Niederkrotenthaler et al., 2020 [28]	Torok et al., 2017 [29]	Sisask et Värnik, 2012 [30]	Mann et al., 2005 [31]
Did the review address a clearly focused question?	Yes	Yes	Yes	Yes	Yes	Yes
Did the authors look for the right type of papers?	Yes	Yes	Yes	Yes	Yes	Yes
Do you think all the important, relevant studies were included?	Yes	Yes	Yes	Yes	No	No
Did the review authors do enough to assess the quality of the included studies?	Yes	Yes	Yes	Unclear	Unclear	Unclear
If the results of the review have been combined, was it reasonable to do so?	Yes	Yes	Yes	Yes	Yes	Yes
What are the overall results of the review?	Yes	Yes	Yes	Yes	Yes	Yes
How precise are the results?	Yes	Yes	Yes	Data was synthesised using a qualitative approach	Data was synthesised using a qualitative approach	Data was synthesised using a qualitative approach
Can the results be applied to the local population?	Yes	Yes	Yes	Yes	Yes	Yes
Were all important outcomes considered?	Yes	Yes	Yes	Yes	Yes	Yes
Are the benefits worth the harm and costs?	Yes	Yes	Yes	Yes	Yes	Yes

**Table 2:** Data extraction form with the characteristics of the six systematic reviews included. Chronological order.

Reference and Year	General Objective	Review Typology	Database Included	Period Covered	Main Findings
Niederkrotenthaler et al., 2022 [26]	Summarise findings from randomised controlled trials on the effects of stories of hope and recovery on individuals with some degree of vulnerability to suicide.	Systematic review and meta-analysis.	PubMed, Scopus, Embase, PsycInfo, Web of Science, and Google Scholar	From inception until 6 September 2021	Exposure to narratives about hope and overcoming suicidal crises appears to have a beneficial effect on people with some vulnerability to suicidal ideation.
Niederkrotenthaler et al., 2021 [27]	Examine the association between portrayals of suicide and suicide attempts in entertainment media and suicidal behaviour in the population.	Systematic review and meta-analysis.	PubMed, Scopus, Embase, PsycInfo, Web of Science, and Google Scholar	From inception until 20 April 2021	The diffusion of suicidal behaviour in the media can increase suicides and suicide attempts among the population. Therefore, they should respect existing guidelines on their safe representation.
Niederkrotenthaler et al., 2020 [28]	Examine the association between reporting on suicides, especially deaths of celebrities by suicide, and subsequent suicides in the general population.	Systematic review and meta-analysis.	PubMed, Scopus, Embase, PsycInfo, Web of Science, and Google Scholar	From inception until 1 September 2019	Guidelines for responsible reporting of suicidal behaviour in the media are the best prevention intervention for the population. They should be more widely applied and promoted.
Torok et al., 2017 [29]	Address key knowledge gaps regarding how mass media campaigns can be optimised to prevent suicide by looking at their global efficacy and mechanisms related to successful outcomes.	Systematic review	PubMed, Scopus, Embase, PsycInfo, Web of Science, Cochrane Library, and Cochrane Central Register of Controlled Trials	From inception until 1 April 2016	Multilevel mass media outreach has positive effects on both suicide rates and suicide attempts. Repeated exposure and community involvement are key aspects of prevention campaigns.
Sisask et Värnik, 2012 [30]	Monitor and provide an overview of the research performed on the roles of the media in suicide prevention in order to find out the possible effects that the media reporting on suicidal behaviours might have on actual suicidality.	Systematic review	PubMed, PsycInfo, and Cochrane Library	From inception until 1 July 2011	Media reports are not representative of official data on suicides. They tend to sensationalise with the exposure of dramatic and very lethal methods which are infrequent in reality.
Mann et al., 2005 [31]	Examine the evidence for the effectiveness of specific suicide-preventive interventions and make recommendations for future prevention programmes and research.	Systematic review	PubMed, PsycInfo, and Cochrane Library	From 1966 to June 2005	Media exposure to suicide as a solution to problems may exacerbate the risk of developing these behaviours.

**Table 3:** Overlap between the systematic reviews and the studies included.

	Authors					
Studies	Niederkrotenthaler et al., 2022 [26]	Niederkrotenthaler et al., 2021 [27]	Niederkrotenthaler et al., 2020 [28]	Torok et al., 2017 [29]	Sisask et Värnik, 2012 [30]	Mann et al., 2005 [31]
Ftanou et al., 2021	x					
Niederkrotenthaler et al., 2020	x					
Till et al., 2020	x					
Niederkrotenthaler et al., 2019			x			
Till et al., 2019	x					
Handley et al., 2018		x				
King et al., 2018	x					
Sinyor et al., 2018			x			
Till et al., 2018			x			
Schmidt, 2017		x				
Till et al., 2017	x					
Arendt et al., 2016	x					
Kontopantelis et al., 2015		x				
Hawton et al., 2014		x				
Matsubayashi et al., 2014				x		
Niederkrotenthaler et al., 2014			x			
Robinson et al., 2014				x		
Robinson et al., 2013				x		
Till et al., 2013				x		
Chen et al., 2010					x	
Jenner et al., 2010				x		
Klimes-Dougan et al., 2010				x		
Niederkrotenthaler et al., 2010			x		x	
Niederkrotenthaler et al., 2009			x			
Klimes-Dougan et al., 2009				x		
Oliver et al., 2008				x		
Daigle et al., 2006				x		
Hegerl et al., 2006				x		
Sudak and Sudak, 2005					x	
Etzersdorfer et al., 1998						x

**Table 4:** Interventions found in the studies reviewed.

	Authors					
Interventions	Niederkrotenthaler et al., 2022 [26]	Niederkrotenthaler et al., 2021 [27]	Niederkrotenthaler et al., 2020 [28]	Torok et al., 2017 [29]	Sisask et Värnik, 2012 [30]	Mann et al., 2005 [31]
Positive messages of hope, resilience, and overcoming adversity.	x	x	x		x	x
Narratives with information on available centres, organisations, and resources.	x			x		x
Promote the attitude of seeking help as an effective mechanism.	x		x	x		
Personal narratives of overcoming suicidal crises.	x					
Specific information aimed at promoting support-seeking oriented towards the male gender.	x		x			
No explicit description of the method used or place/location.		x	x		x	x
No romantic, dramatic, or glorified description of suicide.			x		x	x
No repeated reporting of the same suicide.			x			
Narratives on the availability of treatment for mental disorders.				x		
No portrayal of suicide as inevitable.			x			
Narratives that suicide prevention is possible.				x		
Raise awareness of mental health in the media.				x		x
Work with mental health experts to ensure safe dissemination and exposure.		x				

## Supplementary Materials

The following supporting information can be downloaded at: https://www.mdpi.com/article/10.3390/nursrep13040125/s1, The complete search strategy can be found in Supplementary File S1. Supplementary File S2 contains the studies that met the inclusion criteria.

## References

[1] World Health Organization (1977). The suicide. Notebooks on Public Health No. 59.

[2] Guerrero-Díaz M. (2019). Reflexiones sobre el suicidio desde la mirada histórica. Boletín Psicoevidencias.

[3] Amador Rivera G.H. (2015). Suicidio: Consideraciones históricas. Rev. Médica Paz.

[4] Pan American Health Organization (2022). Suicide Prevention. https://www.paho.org/en/topics/suicide-prevention.

[5] Pompili M., Shrivastava A., Serafini G., Innamorati M., Milelli M., Erbuto D., Ricci F., Lamis D.A., Scocco P., Amore M. (2013). Bereavment after the suicide of a significant other. Indian J. Psychiatry.

[6] Cerel J., Brown M.M., Maple M., Singleton M., Van de Venne J., Moore M., Flaherty C. (2019). How many people are exposed to suicide? Not six. Suicide Life Threat. Behav..

[7] Andriessen K., Rahman B., Draper B., Dudley M., Mitchell P.B. (2017). Prevalence of exposure to suicide: A meta-analysis of population-based studies. J. Psychiatr. Res..

[8] Spanish Government (2015). Sustainable Development Strategy 2030. Ministerio de Derechos Sociales. https://www.mdsocialesa2030.gob.es/agenda2030/documentos/eds-eng-acce.pdf.

[9] World Health Organization (2021). Comprehensive Mental Health Action Plan 2013–2030. World Health Organization. https://www.who.int/publications/i/item/9789240031029.

[10] Wasserman D. (2001). A Stress–Vulnerability Model and the Development of the Suicidal Process: Danuta Wasserman Stress–Vulnerability Model. Suicide.

[11] World Health Organization (2014). Preventing Suicide: A Global Imperative. https://apps.who.int/iris/handle/10665/131056.

[12] Sufrate-Sorzano T., Pérez J., Juárez-Vela R., Garrote-Cámara M., de Viñaspre R.R., Molina-Luque F., Santolalla-Arnedo I. (2022). Umbrella review of nursing interventions NIC for the treatment and prevention of suicidal behavior. Int. J. Nurs. Knowl..

[13] Diez M.T.S., Markina I.C. (2020). La representación del suicidio en la prensa española. Rev. Cienc. Soc..

[14] Fong Y.L. (2021). Reporting on suicide in Malaysia: Problem characterization and solution advocacy by media. KOME.

[15] Phillips D.P. (1974). The influence of suggestion on suicide: Substantive and theoretical implications of the Werther effect. Am. Sociol. Rev..

[16] Niederkrotenthaler T., Voracek M., Herberth A., Till B., Strauss M., Etzersdorfer E., Eisenwort B., Sonneck G. (2010). Role of media reports in completed and prevented suicide: Werther v. Papageno effects. Br. J. Psychiatry.

[17] World Health Organization (2017). Preventing Suicide: A Resource for Media Professionals.

[18] Durán Á., Fernández-Beltrán F. (2020). Responsabilidad de los medios en la prevención del suicidio. Tratamiento informativo en los medios españoles. Prof. Inf..

[19] National Institute of Mental Health (2022). Suicide Prevention. https://www.nimh.nih.gov/health/topics/suicide-prevention.

[20] Ayers J.W., Althouse B.M., Leas E.C., Dredze M., Allem J.P. (2017). Internet searches for suicide following the release of 13 reasons why. JAMA Intern. Med..

[21] Niederkrotenthaler T., Stack S., Till B., Sinyor M., Pirkis J., Garcia D., Rockett I.R.H., Tran U.S. (2019). Association of increased youth suicides in the United States with the release of 13 Reasons Why. JAMA Psychiatry.

[22] Kõlves K., McDonough M., Crompton D., de Leo D. (2018). Choice of a suicide method: Trends and characteristics. Psychiatry Res..

[23] Aromataris E., Fernandez R., Godfrey C., Holly C., Khalil H., Aromataris A.P.E. (2014). Methodology for jbi umbrella reviews. The Joanna Briggs Institute Reviewers Manual.

[24] Gates M., Gates A., Pieper D., Fernandes R.M., Tricco A.C., Moher D., E Brennan S., Li T., Pollock M., Lunny C. (2022). Reporting guideline for overviews of reviews of healthcare interventions: Development of the PRIOR statement. BMJ.

[25] (2022). CASP Checklists. Critical Appraisal Skills Programme. https://casp-uk.net/casp-tools-checklists/.

[26] Niederkrotenthaler T., Till B., Kirchner S., Sinyor M., Braun M., Pirkis J., Tran U.S., Voracek M., Arendt F., Ftanou M. (2022). Effects of media stories of hope and recovery on suicidal ideation and help-seeking attitudes and intentions: Systematic review and meta-analysis. Lancet Public Health.

[27] Niederkrotenthaler T., Kirchner S., Till B., Sinyor M., Tran U.S., Pirkis J., Spittal M.J. (2021). Systematic review and meta-analyses of suicidal outcomes following fictional portrayals of suicide and suicide attempt in entertainment media. EClinicalMedicine.

[28] Niederkrotenthaler T., Braun M., Pirkis J., Till B., Stack S., Sinyor M., Tran U.S., Voracek M., Cheng Q., Arendt F. (2020). Association between suicide reporting in the media and suicide: Systematic review and meta-analysis. BMJ.

[29] Torok M., Calear A., Shand F., Christensen H. (2017). A systematic review of mass media campaigns for suicide prevention: Understanding their efficacy and the mechanisms needed for successful behavioral and literacy change. Suicide Life Threat. Behav..

[30] Sisask M., Värnik A. (2012). Media roles in suicide prevention: A systematic review. Int. J. Environ. Res. Public Health.

[31] Mann J.J., Apter A., Bertolote J., Beautrais A., Currier D., Haas A., Hegerl U., Lonnqvist J., Malone K., Marusic A. (2005). Suicide prevention strategies: A systematic review: A systematic review. JAMA.

[32] Hennessy E.A., Johnson B.T. (2020). Examining overlap of included studies in meta-reviews: Guidance for using the corrected covered area index. Res. Synth. Methods.

[33] Donovan J., Boyd D. (2021). Stop the presses? Moving from strategic silence to strategic amplification in a networked media ecosystem. Am. Behav. Sci..

[34] (2022). Action Alliance. National Recommendations for Depicting Suicide. https://theactionalliance.org/messaging/entertainment-messaging/national-Recommendations.

[35] Antebi L., Carmichael V., Whitley R. (2020). Assessing Adherence to Responsible Reporting of Suicide Guidelines in the Canadian News Media: A 1-year Examination of Day-to-day. Can. J. Psychiatry.

[36] Sinyor M., Schaffer A., Heisel M.J., Picard A., Adamson G., Cheung C.P., Katz L.Y., Jetly R., Sareen J. (2018). Media guidelines for reporting on suicide: 2017 update of the Canadian psychiatric association policy paper. Can. J. Psychiatry.

[37] World Health Organization (2008). Preventing Suicide: A Resource for Media Professionals.

[38] Pan American Health Organization (2018). Suicide Prevention: A Resource for Media Professionals. Update.

[39] Acosta-Artiles F.J., Rodríguez-Caro C.J., Cejas-Méndez M.R. (2017). Noticias sobre suicidio en los medios de comunicación. Recomendaciones de la OMS. Rev. Española Salud Pública.

[40] Armstrong G., Vijayakumar L., Cherian A., Krishnaswamy K., Pathare S. (2021). Indian media professionals’ perspectives regarding the role of media in suicide prevention and receptiveness to media guidelines: A qualitative study. BMJ Open.

[41] Sufrate-Sorzano T., Jiménez-Ramón E., Garrote-Cámara M.E., Gea-Caballero V., Durante A., Júarez-Vela R., Santolalla-Arnedo I. (2022). Health plans for suicide prevention in Spain: A descriptive analysis of the published documents. Nurs. Rep..

[42] Working Group of the Suicide Prevention Plan in La Rioja (2018). Suicide Prevention Plan in La Rioja.

[43] Schmidtke A., Häfner H., Diekstra R.F.W., Maris R., Platt S., Schmidtke A., Sonneck G. (1989). Public Attitudes towards and Effects of the Mass Media on Suicidal and Deliberate Self-Harm Behavior. Suicide and Its Prevention, The Role of Attitude and Imitation.

