# Supplementary material for: Media Exposure of Suicidal Behaviour: An Umbrella Review
Source: Nurs Rep. 2023 Oct 25;13(4):1486–99. doi: 10.3390/nursrep13040125 (PMC10660843; doi:10.3390/nursrep13040125)
Supplement: Supplementary file 1 [file nursrep-13-00125-s001.zip › nursrep-2587782-supplementary.pdf]

SUPPLEMENTARY FILE S1. Search strategy for the eight electronic databases.

**PUBMED. Date of Search: 13/02/2023. Number of results: 204**		
**#**	**Search string**	**Results**
1	"Suicide"[Mesh] OR "Suicide, Completed"[Mesh] OR "Suicidal Ideation"[Mesh] OR "Suicide, Attempted"[Mesh] OR Suicid*[tiab] OR Parasuicid*[tiab] OR "suicidal behavior*"[tiab] OR "suicidal behaviour*"[tiab] OR "completed suicide"[tiab] OR "Suicidal Ideation"[tiab] OR "attempted suicide"[tiab]	111,311
2	""Communication Media"[tiab] OR news[tiab] OR newspaper*[tiab] OR "newspaper article*"[tiab] OR periodical*[tiab] OR "Newspaper Article"[Publication Type] OR "News"[Publication Type] OR "Video-Audio Media"[Publication Type] OR Audiovisual Media[tiab] OR Audio-Visual Media[tiab] OR "videotape recording*"[tiab] OR "Audiovisual Aids"[Mesh] OR "audiovisual aid*"[tiab] OR "Radio"[Mesh] OR "Television"[Mesh] OR television*[tiab] OR "Telecommunications"[Mesh] OR telecommunication*[tiab] OR campaign*[tiab] OR advert*[tiab] OR "mass medium*"[tiab] OR "audiovisual equipment*"[tiab]	642,103
3	meta-analysis OR "meta analy*" OR metanaly* OR metaanaly* OR ((systematic* OR evidence*) AND (review* OR overview*)) OR "meta analysis" OR (("multiple treatment*" OR "indirect" OR "mixed") AND "comparison*") OR "systematic review" OR "reference list*" OR bibliograph* OR "hand search*" OR "manual search*" OR "relevant journals" OR "search strategy" OR "search criteria" OR "systematic search" OR "study selection" OR "data extraction" OR (search* AND literature) OR (("multiple treatment*" OR indirect OR mixed) AND comparison*) OR medline OR pubmed OR cochrane OR embase OR psychlit OR psyclit OR psychinfo OR psycinfo OR cinahl OR "science citation index" OR "Cochrane Database Syst Rev"[jour] OR "meta analysis as topic"[MeSH Terms] OR "systematic reviews as topic"[MeSH Terms] OR "meta analysis"[Publication Type] OR "systematic review"[Publication Type]	1,239,647
4	#1 AND #2 AND #3	204
**CINAHL & PsycInfo. Date of Search: 13/02/2023. Number of results: 2.981**		
**#**	**Search string**	**Results**
1	(MH "Suicide+") OR (MH "Suicide, Attempted") OR (MH "Suicidal Ideation") OR Suicid* OR Parasuicid* OR "suicidal behavior*" OR "suicidal behaviour*" OR "completed suicide" OR "Suicidal Ideation" OR "attempted suicide"	130,694
2	" Communication Media" OR news OR newspaper* OR "newspaper article*" OR periodical* OR Audiovisual Media OR Audio-Visual Media OR "videotape recording*" OR "audiovisual aid*" OR television* OR telecommunication* OR campaign* OR advert* OR "mass medium*" OR "audiovisual equipment*" OR (MH "Communications Media+") OR (MH "News+") OR (MH "Television") OR (MH "Audiorecording") OR (MH "Radio") OR (MH "Videorecording+")	932,051
3	(MH "Cochrane Library") OR (MH "Literature Review+") OR meta-analysis OR "meta analy*" OR metanaly* OR metaanaly* OR ((systematic* OR evidence*) AND (review* OR overview*)) OR "meta analysis" OR (("multiple treatment*" OR "indirect" OR "mixed") AND "comparison*") OR "systematic review" OR "reference list*" OR bibliograph* OR "hand search*" OR "manual search*" OR "relevant journals" OR "search strategy" OR "search criteria" OR "systematic search" OR "study selection" OR "data extraction" OR (search* AND literature) OR (("multiple treatment*" OR indirect OR mixed) AND comparison*) OR medline OR pubmed OR cochrane OR embase OR psychlit OR psyclit OR psychinfo OR psycinfo OR cinahl OR "science citation index"	5,495,906
4	S1 AND S2 AND S3	2,981
**WEB OF SCIENCE. Date of Search: 13/02/2023. Number of results: 212**		
**#**	**Search string**	**Results**
1	ALL=(meta-analysis OR "meta analy*" OR metanaly* OR metaanaly* OR ((systematic* OR evidence*) AND (review* OR overview*)) OR "meta analysis" OR (("multiple treatment*" OR "indirect" OR "mixed") AND "comparison*") OR "systematic review" OR "reference list*" OR bibliograph* OR "hand search*" OR "manual search*" OR "relevant journals" OR "search strategy" OR "search criteria" OR "systematic search" OR "study selection" OR "data extraction" OR (search* AND literature) OR (("multiple treatment*" OR indirect OR mixed) AND comparison*) OR medline OR pubmed OR cochrane OR embase OR psychlit OR psyclit OR psychinfo OR psycinfo OR cinahl OR "science citation index")	1,750,831
2	ALL=("Communication Media" OR news OR newspaper* or "newspaper article*" OR periodical* OR Audiovisual Media OR Audio-Visual Media OR "videotape recording*" OR "audiovisual aid*" OR television* OR telecommunication* OR campaign* OR advert* OR "mass medium*" OR "audiovisual equipment*")	1,583,167
3	ALL=(Suicid* OR Parasuicid* OR "suicidal behavior*" OR "suicidal behaviour*" OR "completed suicide" OR "Suicidal Ideation" OR "attempted suicide")	119,517
4	#3 AND #2 AND #1	212

EMBASE. Date of Search: 13/02/2023. Number of results: 936		
#	Search string	Results
1	'suicide attempt'/exp OR 'suicide attempt'	39,904
2	'suicidal behavior'/exp OR 'suicidal behavior'	127,463
3	'suicide'/exp OR suicide	133,452
4	'suicidal ideation'/exp OR 'suicidal ideation'	33,561
5	suicid* OR parasuicid* OR 'suicidal behavior*' OR 'suicidal behaviour*' OR 'completed suicide'/exp OR 'completed suicide' OR 'suicidal ideation'/exp OR 'suicidal ideation' OR 'attempted suicide'/exp OR 'attempted suicide'	162,909
6	#1 OR #2 OR #3 OR #4 OR #5	163,894
7	'publication'/exp OR publication	461,239
8	'audiovisual equipment'/exp OR 'audiovisual equipment'	122,456
9	'mass medium'/exp OR 'mass medium'	20,467
10	'communication media' OR 'news'/exp OR news OR newspaper* OR 'newspaper article*' OR periodical* OR 'audiovisual media'/exp OR 'audiovisual media' OR (audiovisual AND ('media'/exp OR media)) OR 'audio-visual media' OR ('audio visual' AND ('media'/exp OR media)) OR 'videotape recording*' OR 'audiovisual aid*' OR television* OR telecommunication* OR campaign* OR advert* OR 'mass medium*' OR 'audiovisual equipment*'	692,341
11	#7 OR #8 OR #9 OR #10	1,133,877
12	'systematic review (topic)'/exp OR 'systematic review (topic)' OR 'systematic review'/exp OR 'systematic review'	493,538
13	'meta analy*':ab,ti OR metanaly*:ab,ti OR metaanaly*:ab,ti OR ((systematic*:ab,ti OR evidence*:ab,ti) AND (review*:ab,ti OR overview*:ab,ti)) OR 'meta analysis':ab,ti OR (('multiple treatment*':ab,ti OR 'indirect':ab,ti OR 'mixed':ab,ti) AND 'comparison*':ab,ti) OR 'systematic review':ab,ti OR 'reference list*':ab,ti OR bibliograph*:ab,ti OR 'hand search*':ab,ti OR 'manual search*':ab,ti OR 'relevant journals':ab,ti OR 'search strategy':ab,ti OR 'search criteria':ab,ti OR 'systematic search':ab,ti OR 'study selection':ab,ti OR 'data extraction':ab,ti OR (search*:ab,ti AND literature:ab,ti) OR (('multiple treatment*':ab,ti OR indirect:ab,ti OR mixed:ab,ti) AND comparison*:ab,ti) OR medline:ab,ti OR pubmed:ab,ti OR cochrane:ab,ti OR embase:ab,ti OR psychlit:ab,ti OR psyclit:ab,ti OR psychinfo:ab,ti OR psycinfo:ab,ti OR cinahl:ab,ti OR 'science citation index':ab,ti	1,252,721
14	#12 OR #13	1,314,708
15	#6 AND #11 AND #14	936

COCHRANE. Date of Search: 13/02/2023. Number of results: 115		
#	Search string	Results
1	MeSH descriptor: [Systematic Reviews as Topic] explode all trees	54
2	MeSH descriptor: [Mass Media] explode all trees	2,100
3	MeSH descriptor: [Suicide] explode all trees	1,803
4	MeSH descriptor: [Suicide, Attempted] explode all trees	577
5	meta-analysis OR “meta analy*” OR metanaly* OR metaanaly* OR ((systematic* OR evidence*) AND (review* OR overview*)) OR "meta analysis" OR (("multiple treatment*" OR "indirect" OR "mixed") AND "comparison*") OR "systematic review" OR “reference list*” OR bibliograph* OR “hand search*” OR “manual search*” OR “relevant journals” OR “search strategy” OR “search criteria” OR “systematic search” OR “study selection” OR “data extraction” OR (search* AND literature) OR ((“multiple treatment*” OR indirect OR mixed) AND comparison*) OR medline OR pubmed OR cochrane OR embase OR psychlit OR psyclit OR psychinfo OR psycinfo OR cinahl OR “science citation index”	1.985.432
6	"Communication Media" OR news OR newspaper* OR "newspaper article*" OR periodical* OR Audiovisual Media OR Audio-Visual Media OR "videotape recording*" OR "audiovisual aid*" OR television* OR telecommunication* OR campaign* OR advert* OR "mass medium*" OR "audiovisual equipment*"	17.857
7	Suicid* OR Parasuicid* OR “suicidal behavior*” OR “suicidal behaviour*” OR “completed suicide” OR "Suicidal Ideation" OR “attempted suicide”	8.388
8	#7 OR #4 OR #3	8,388
9	#5 OR #1	1.985.432
10	#6 OR #2	18.316
11	#9 AND #10 AND #8	115

**SCOPUS. Date of Search: 13/02/2023. Number of results: 50**		
#	Search string	Results
1	TITLE-ABS-KEY (meta-analysis OR "meta analy*" OR metanaly* OR metaanaly* OR ((systematic* OR evidence*) AND (review* OR overview*)) OR "meta analysis" OR (("multiple treatment*" OR "indirect" OR "mixed") AND "comparison*") OR "systematic review" OR "reference list*" OR bibliograph* OR "hand search*" OR "manual search*" OR "relevant journals" OR "search strategy" OR "search criteria" OR "systematic search" OR "study selection" OR "data extraction" OR (search* AND literature) OR (("multiple treatment*" OR indirect OR mixed) AND comparison*) OR medline OR pubmed OR cochrane OR embrace OR psychic OR psychiat OR psychic OR psycho OR cineol OR "science citation index")	1.889.135
2	TITLE-ABS-KEY ("Communication Media" OR news OR newspaper* OR "newspaper article*" OR periodical* OR audiovisual AND media OR audio-visual AND media OR "videotape recording*" OR "audiovisual aid*" OR television* OR telecommunication* OR campaign* OR advert* OR "mass medium*" OR "audiovisual equipment*")	88.227
3	TITLE-ABS-KEY (suicid* OR parasuicid* OR "suicidal behavior*" OR "suicidal behaviour*" OR "completed suicide" OR "Suicidal Ideation" OR "attempted suicide")	172.632
4	S1 AND S2 AND S3	50
**GOOGLE SCHOLAR. Date of Search: 13/02/2023. Number of results: 22**		
1	"systematic review" "Communication Media" "suicidal behavior" "Suicidal Ideation" "attempted suicide"	22

SUPPLEMENTARY FILE S2. Studies that might appear to meet the inclusion criteria.

Reference and year	Main reason for exclusion
Szumilas et Kutcher, 2009	No systematic review
Mee-Huong et al., 2014	Out of scope
Hernández et al., 2015	Out of scope
Larsen et al., 2016	Out of scope
Quigley et al., 2016	Out of scope
Hoffman et al., 2017	No systematic review
McClatchey et al., 2017	Out of scope
Franco-Martin et al., 2018	Out of scope
McClatchey et al., 2018	Out of scope
Mishara et Dargis, 2019	No systematic review
Saquete et al., 2019	Out of scope
Bada et Clayton, 2020	No systematic review
Biernsesser et al., 2020	No systematic review
Evans et Abrahamson, 2020	Out of scope
Sanchez et al., 2020	Out of scope
April et al., 2021	Out of scope
Dabkowski et Porter., 2021	Out of scope
Sonke et al., 2021	Out of scope
Brennan et al., 2022	Out of scope
